# Flexible Seven-in-One Microsensor Embedded in High-Pressure Proton Exchange Membrane Water Electrolyzer for Real-Time Microscopic Monitoring

**DOI:** 10.3390/s23125489

**Published:** 2023-06-10

**Authors:** Chi-Yuan Lee, Chia-Hung Chen, Hsian-Chun Chuang, Shan-Yu Chen, Yu-Chen Chiang

**Affiliations:** 1 Department of Mechanical Engineering, Yuan Ze Fuel Cell Center, Yuan Ze University, Taoyuan 32003, Taiwan; 2 Homytech Global Co., Ltd., Taoyuan 33464, Taiwan

**Keywords:** high-pressure PEMWE, MEMS, flexible seven-in-one microsensor

## Abstract

The voltage, current, temperature, humidity, pressure, flow, and hydrogen in the high-pressure proton exchange membrane water electrolyzer (PEMWE) can influence its performance and life. For example, if the temperature is too low to reach the working temperature of the membrane electrode assembly (MEA), the performance of the high-pressure PEMWE cannot be enhanced. However, if the temperature is too high, the MEA may be damaged. In this study, the micro-electro-mechanical systems (MEMS) technology was used to innovate and develop a high-pressure-resistant flexible seven-in-one (voltage, current, temperature, humidity, pressure, flow, and hydrogen) microsensor. It was embedded in the upstream, midstream, and downstream of the anode and cathode of the high-pressure PEMWE and the MEA for the real-time microscopic monitoring of internal data. The aging or damage of the high-pressure PEMWE was observed through the changes in the voltage, current, humidity, and flow data. The over-etching phenomenon was likely to occur when this research team used wet etching to make microsensors. The back-end circuit integration was unlikely to be normalized. Therefore, this study used lift-off process to further stabilize the quality of the microsensor. In addition, the PEMWE is more prone to aging and damage under high pressure, so its material selection is very important.

## 1. Introduction

Due to increasingly serious global warming, the fifth assessment report of the Intergovernmental Panel on Climate Change (IPCC) pointed out [1] that the temperature increased by 0.85 °C from 1880 to 2012. The man-induced total emission of greenhouse gases increased continuously between 1970 and 2010. The CO_2_ emitted from fossil fuel combustion and industry accounted for 78% of the total emission increment of greenhouse gases. According to the 2022 global carbon budget published by the Global Carbon Project [2], the global total CO_2_ emission in 2022 is estimated at 40.6 billion tons. If this data is maintained, the global average temperature rise will exceed 1.5 °C within 9 years with 50% probability. In order to reduce the emission of greenhouse gases, the world is actively seeking new energy which can replace fossil energy, and the fact that hydrogen energy is characterized by stable current, low noise, high efficiency, and greenhouse-gas-free power generation process makes the first choice for the development of new energy in the world.

The proton exchange membrane water electrolyzer (PEMWE) is the most advanced water electrolyzer type at present. Compared with traditional water electrolysis technology, the PEMWE has the advantages of lower corrosivity, relatively lower working temperature, and higher energy conversion efficiency [3,4]. Bareiß et al. [5] indicated, that with appropriate storage capacity, the surplus hydrogen produced can be reconverted and used as fuel for fuel cell electric vehicles. PEMWE has a crucial role to play in renewable energy. Kang et al. [6] used a novel four-wire sensing technology to sense the internal voltage loss of PEMWE in operation. The ohmic resistance of the anode catalyst layer decays exponentially with the current density, and varies with the working temperature. The ohmic resistance of the cathode catalyst layer can be kept constant because of the strong electrical conductivity of Pt/C in the cathode catalyst layer. It has nothing to do with operating conditions such as working current density and temperature. Liu et al. [7] found that high voltage uniformity in the dynamic process is the key to the durability of the cell stack. The voltage uniformity will be affected by temperature, current, and current increasing rate. Shin et al. [8] found that the mass flow rate of hydrogen-containing discharged water varies with current density and temperature. However, hydrogen-containing discharged water is less influenced by temperature than current density. Ogumerem et al. [9] mentioned that membrane degradation is induced by the thermal, chemical, and mechanical stresses during the operation of PEMWE. The thermal stress has the greatest impact because high working temperatures make the polymer film easier to deform. The chemical degradation induced by free radicals is increased in high-pressure operation. Chandesris et al. [10] performed single cell degradation experiments, demonstrating that most of the membrane degradation occurred on the cathode side. They found that the degradation rate was low when the current density was low. It increased with increasing current density, reached a maximum, and then decreased. Fouda-Onana et al. [11] found that, in electrochemical and physicochemical analyses, high temperature had a higher contribution to membrane degradation than current density. Tjarks et al. [12] indicated that adjusting the pressure of the electrolyzer to the optimal working pressure can reduce the energy demand of the whole system, to achieve the best efficiency, and its optimal working pressure depends on the current density. If the pressure exceeds the optimal working pressure continuously, the seepage loss increases, so that the energy conversion efficiency of the overall system decreases. Upadhyay et al. [13] found that the performance of PEMWE in the area of high current density could be degraded by increasing the cathode pressure. Diéguez et al. [14] found that natural convection control heat loss accounts for a very large part of the generated heat energy. On the other hand, a very large part of energy is dissipated in the form of heat; the electrolytic tank can be kept at an appropriate temperature to reduce the battery overvoltage, so that higher conversion efficiency can be achieved. Selamet et al. [15] indicated that the performance of the cell stack is enhanced at high temperatures because the temperature rise increases the reaction kinetics, reduces the activation energy barrier, and enhances the ionic conductivity of membrane. Scheepers et al. [16] found that the optimum temperature approaches the ambient temperature at low current density, and increases with the current density, while the low temperatures can improve the durability of PEMWE. Bender et al. [17] indicated that the performance deviation usually increases with the current density, and the deviation is higher when the battery temperature is lower, meaning it is more difficult to implement reproducible conditions at 60 °C; it may be related to the water flow rate through the battery and relevant thermal insulation in the measuring process. The temperature has a significant impact on the performance of the PEMWE. The flow rate has no significant impact, but the gas removal rate can be increased by increasing the flow rate, so as to improve the performance of the PEMWE [18]. Additionally, the water can be used as the coolant of cell stack; if the flow is lower than the critical value, the cell stack may be overheated [19]. To ensure the water supply to the electrolytic tank and the removal of product gas, although the water supply was required only on the anode side, Maier et al. [20] used flow field on the cathode side to promote hydrogen and remove excess water.

This study used MEMS technology to innovate and develop a high-pressure-resistant flexible seven-in-one microsensor. It was embedded in the upstream, midstream and downstream of the anode and cathode of the high-pressure PEMWE and the MEA for the real-time microscopic monitoring of seven important physical quantities. Widera et al. [21] proposed that the hydrogen cycle, including production, storage, and utilization, is widely studied. Although there are various manufacturing technologies (such as biological production, steam reforming, etc.), water electrolyzers are the focus of research and development of energy-related technologies because they can be combined with renewable energy. At present, the physical quantities of the PEMWE are all obtained from external measurement and simulation, but the actual operation of the PEMWE is very important. The internal data (voltage, current, temperature, humidity, pressure, flow, and hydrogen) will all affect each other and affect the data of the PEMWE. Therefore, our team uses MEMS technology to develop a flexible seven-in-one microsensor. Compared with the previous study [22], this study increased from embedding three sets of sensors to seven sets of sensors and carried out real-time microscopic monitoring of the upstream, midstream, and downstream of the anode and cathode of the high-pressure PEMWE and the MEA.

## 2. High-Pressure PEMWE

The high-pressure PEMWE used in this study included the MEA and bipolar plate runner designs, which are combined with the collector plate designed in this study, referring to the assembly procedures of related techniques and the improvements for enhancing the performance.

### 2.1. High-Pressure PEMWE Design

**Figure 1:**
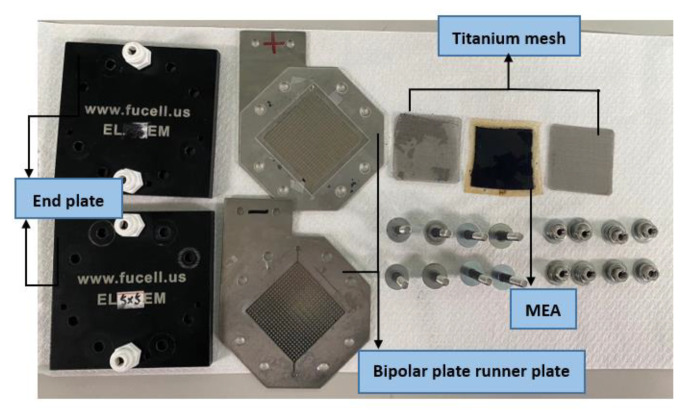
High-pressure PEMWE.

The overall dimension of the high-pressure PEMWE are 112 mm × 112 mm × 140 mm, and the reaction area is 50 × 50 mm^2^. The overall parts include two end plates, two bipolar runner plates, a titanium mesh, and a MEA, as shown in Figure 1. The collector plate and runner plate of this high-pressure PEMWE are designed as an integer; the material is improved to metal material, resulting in high mechanical strength and electrochemical resistance. The two holes in the anode terminal are the pure water inlet and pure water outlet. The oxygen is discharged through the pure water outlet during operation. The two holes in the cathode terminal discharge hydrogen.

### 2.2. End Plate

**Figure 2:**
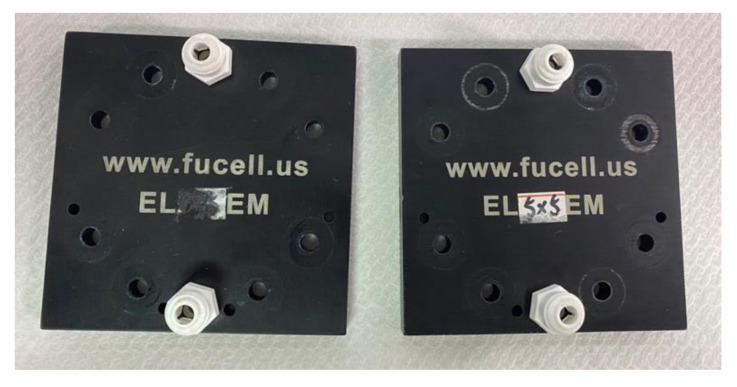
Titanium end plate (90 mm × 90 mm × 10 mm).

The size of titanium end plate for the high-pressure PEMWE is 90 mm × 90 mm × 10 mm, as shown in Figure 2. It supplies uniform pressure to the water electrolyzer to fix it. It is equipped with eight small holes and bolts to fix its electrical conductivity, stabilize the electrochemical reaction state, and prevent the leakage of gases and DI water.

### 2.3. MEA

**Figure 3:**
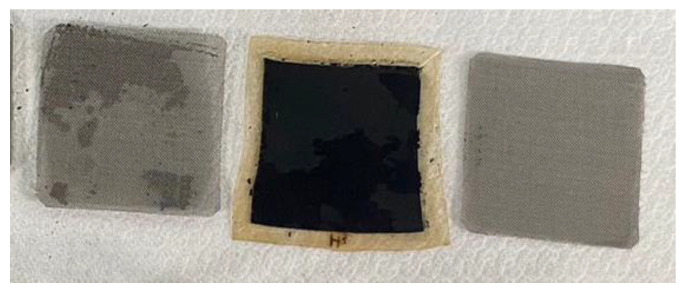
MEA (50 mm × 50 mm).

The design and material of the MEA are the major factors which influence the overall performance of the high-pressure PEMWE. The MEA is the region for electrochemical reaction, it is mainly composed of the proton exchange membrane (PEM) located in the middle of the structure, a longitudinally symmetrical catalyst layer, and two titanium meshes, as shown in Figure 3.

The main function of the PEM is to prevent the DI water from flowing from the anode to the cathode, and to prevent the ions and electrons other than protons from passing through the membrane material. Therefore, the PEM must have good mechanical strength and electrochemical resistance, as well as good proton penetration. The MEA used in this study was made by using the nanocatalyst spray coating technique. The core technology aims to increase the catalyst efficiency and reduce the catalyst consumption.

**Figure 4:**
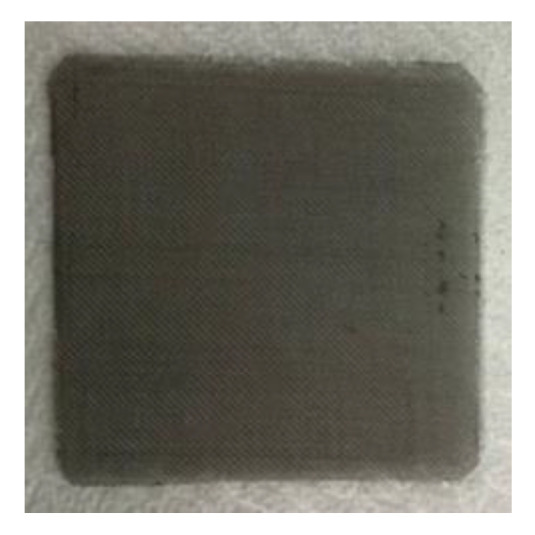
Titanium mesh (50 mm × 50 mm).

In terms of gas diffusion layer, the original carbon cloth was substituted by titanium mesh, as shown in Figure 4. It acted as a bridge between the MEA and bipolar plate. Its structure affected gas permeation, water accumulation, and electron conduction. The titanium mesh enhanced waterproofness and moisture permeability for its high porosity, and the titanium metal surface was likely to oxidize to form TiO_2_. The electron conductivity of titanium mesh was reduced, and the contact impedance was lower than that of carbon cloth, so that the performance of the high-pressure PEMWE was enhanced.

### 2.4. Bipolar Plate Runner Design

**Figure 5:**
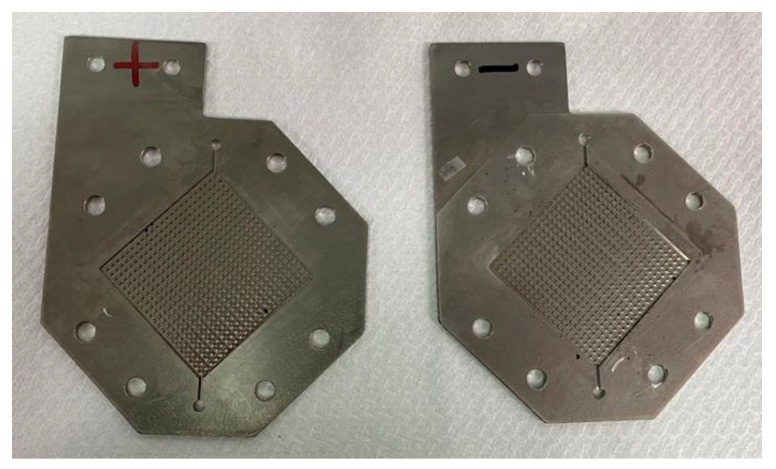
Collector and bipolar runner plates.

The bipolar runner plate and collector plate in this study were integrated, as shown in Figure 5. They were made of titanium material with strong electrical conductivity, corrosion resistance, high conductivity, and good thermal conductivity. The material cost was lower than that of a graphite plate.

**Figure 6:**
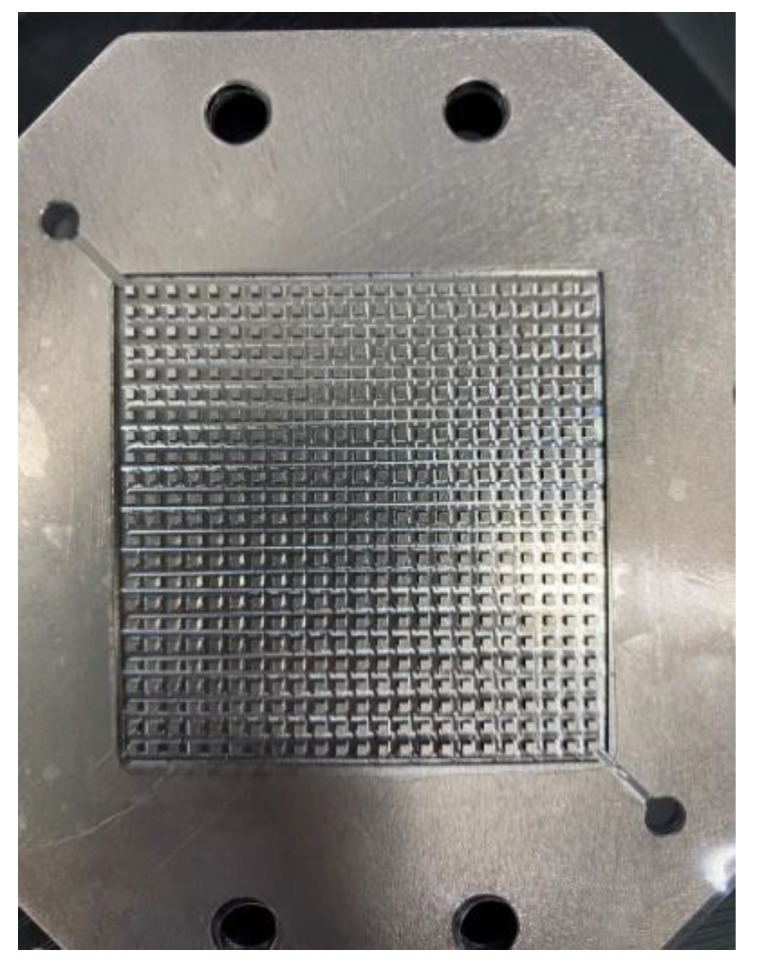
Stereogram of column-like flow channel design runner width (12 mm).

The PEMWE flow channel design was mainly divided into serpentine flow channel, grid flow channel, and column-like flow channel. The purpose was to uniformly distribute the fluid on the reaction area of the MEA, and to take the products of electrochemical reaction (hydrogen, oxygen, and water) away smoothly. To ensure even temperature distribution over the flow channel structure of the high-pressure PEMWE, and to avoid the flow channel being blocked, the column-like flow channel design was used, as shown in Figure 6. The fluid could flow through uniformly, and the gases could be taken away rapidly, so that the hydrogen and oxygen could be discharged quickly, and the reaction area of the high-pressure PEMWE would not be reduced by the gases in the flow channel.

## 3. The Principle of the High-Voltage Flexible Seven-in-One Microsensor

### 3.1. The Principle of Microvoltage Sensor

The microvoltage sensor in this research uses a metal probe and miniaturizes it as a microvoltage sensor. The material is gold (Au). To ensure that its voltage will not be affected by other parts, FUJIFILM Electronic Materials (Taiwan Co., Ltd., Hsin-Chu, Taiwan) is used, Inc. LTC^®^ 9320 liquid polyimide covers the part of the middle extension wire to ensure that the sensing voltage is the local area being measured.

### 3.2. The Principle of Microcurrent Sensor

The microcurrent sensor is the same as the microvoltage sensor. It uses Fujifilm Electronic Materials (Taiwan Co., Ltd., Hsin-Chu, Taiwan) LTC^®^ 9320 to cover the middle extension wire, and penetrates into the cathode and anode of the high-voltage proton exchange membrane water electrolyzer and the inside of the MEA membrane. The current is drawn to the measuring instrument to measure the local current of the high-voltage proton exchange membrane water electrolyzer.

### 3.3. The Principle of Microtemperature Sensor

This research uses a resistance temperature detector (Resistance Temperature Detector, RTD), which uses the resistance change caused by the metal when the temperature changes as the temperature-sensing principle. The front part adopts a snake structure to increase its resistance. Gold (Au) is used as a temperature-sensing resistance material due to its stable chemical properties, simple manufacturing process, and high linearity.

### 3.4. The Principle of Microhumidity Sensor

The microhumidity sensor uses a resistive snake-type microhumidity sensor, and uses the hydrophilic polymer material Fujifilm Electronic Materials (Taiwan Co., Ltd., Hsin-Chu, Taiwan) LTC^®^ 9305 as the humidity-sensing material. The polymer material of the microhumidity sensor has a low dielectric constant (about 3–4), and the dielectric constant of water is 80. When the volume of the material increases with the amount of moisture absorbed, the adsorption circuit resistance will also increase.

### 3.5. The Principle of Microflow Sensor

The measurement method used in this research is a snake-shaped hot-wire microflow sensor. The hot-wire microflow sensor has the advantages of small size, low power consumption, high sensitivity, and high precision, so it is very suitable for this research. It uses gold (Au) with good physical and chemical properties and a simple manufacturing process as the electrode material. The power supply is used to provide a constant voltage to make the sensing end generate a certain amount of heat. When a fluid passes through the heat source, the heat source is taken away. Additionally, the temperature of the electrode is lowered, and the temperature change will cause its resistance to change. According to Ohm’s law, when the resistance changes under the condition of constant voltage, the current will also change, and the value is measured using this principle.

### 3.6. The Principle of Micropressure Sensor

In this study, the capacitive micropressure sensor is a parallel plate capacitor with a sandwich structure. The upper and lower sides of the capacitive micropressure sensor are two parallel interlayer electrodes, and a nonconductive dielectric layer (Dielectric layer) is sandwiched between the two parallel electrodes.

### 3.7. The Principle of Microhydrogen Sensor

The microhydrogen sensor in this study uses a semiconductor sensor, and the main principle is to use the reducing gas and the oxygen on the surface of the gas-sensitive film. Because oxygen atoms (O) are adsorbed on the surface of the gas-sensitive film, it is easy to take away electrons inside the material to form oxygen ions (O-), which reduces the electrons inside the gas-sensitive film and causes the resistance-sensing material to rise; when exposed to reducing gases (such as CO, H_2_, etc.) it will reduce the overall resistance of the sensing material.

## 4. Microscopic Monitoring of High-Pressure PEMWE

**Figure 7:**
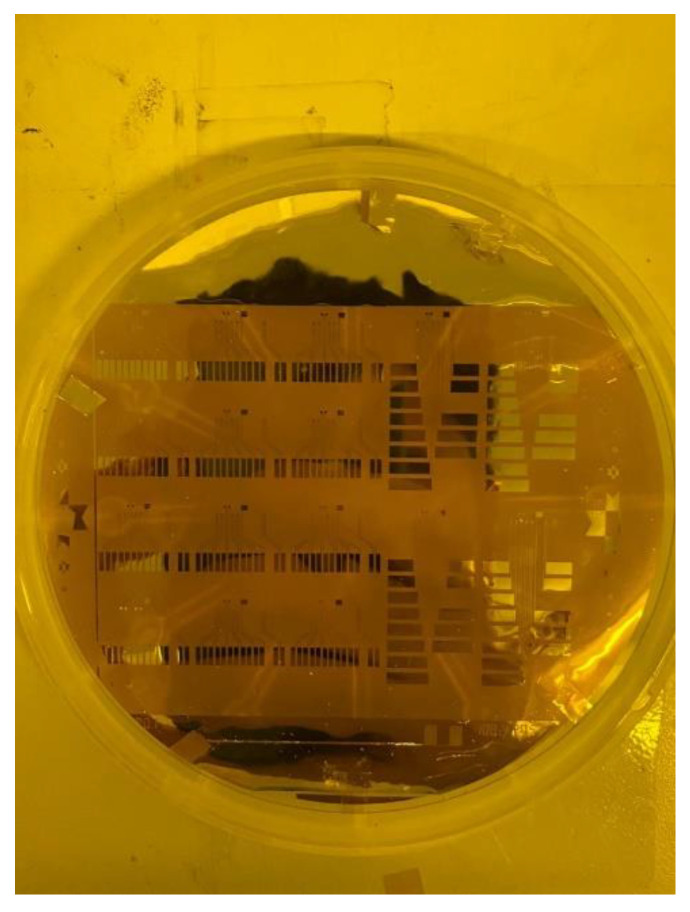
Lift-off process completed.

Our laboratory uses “gold etchant” for etching, and acetone is used for the lift-off part. The wet etching method has the risk of over-etching, and the cost of the etching solution is also higher than that of acetone. Therefore, this experiment adopts the lift-off process; the test piece after the lift-off process is completed is shown in Figure 7.

## 5. Microscopic Monitoring of High-Pressure PEMWE

To prevent the overflow of water in the high-pressure PEMWE, the nuts must be diagonally tightened to 6 Nm with a torque wrench. The torsion of each nut must also be consistent, otherwise the uneven stress may lead to deformation fractures of the bipolar plate and the collector plate. Finally, the lbs is locked up, more than two cycles is preferred, and the application of force is ensured.

### 5.1. Real-Time Microscopic Monitoring of High-Pressure PEMWE

**Figure 8:**
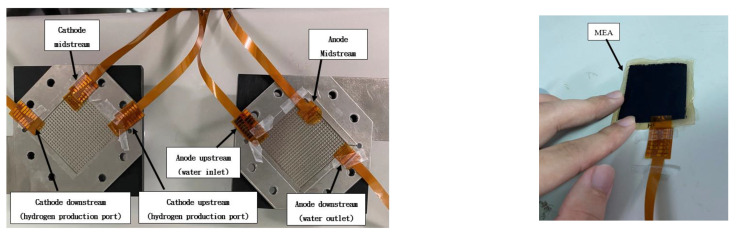
High-voltage-resistant flexible seven-in-one microsensor embedded with anode and cathode and MEA.

**Figure 9:**
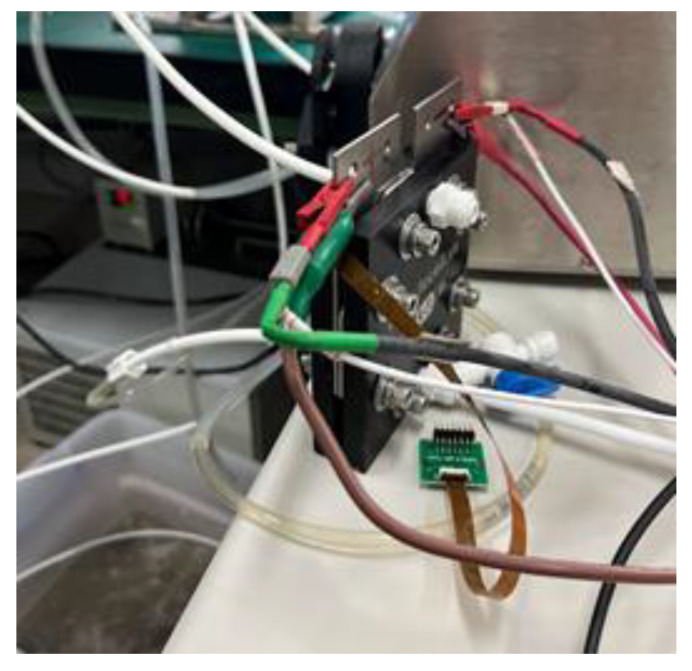
Assembly diagram of high-voltage flexible seven-in-one microsensor embedded in high-voltage PEMWE.

In this study, an innovatively developed flexible seven-in-one microsensor is implanted upstream, midstream, and downstream of the anode and cathode of high-pressure PEMWE and MEA. Seven sets of high-pressure-resistant flexible seven-in-one microsensors are implanted, as shown in Figure 8. An assembly diagram of high-voltage flexible seven-in-one microsensor embedded in the high-voltage PEMWE is shown in Figure 9. The high-pressure-resistant flexible seven-in-one microsensor, power supply, peristaltic pump, and high-accuracy acquisition equipment NI PXI 2575 data acquisition unit were used for 100 h microscopic monitoring.

### 5.2. High-Pressure PEMWE Testing Environment

The high-pressure PEMWE was activated before the 100 h real-time micromonitoring. The activation as performed with 2.6 V constant voltage, and the operation continued until the current reached 25 A and decreased to stabilization. The 100 h real-time microscopic monitoring of the high-pressure PEMWE was performed with DI water at temperature 25 °C and flow velocity 100 mL/min and by controlling the cathode pressure at 3 bar.

### 5.3. Voltage in High-Pressure PEMWE

**Figure 10:**
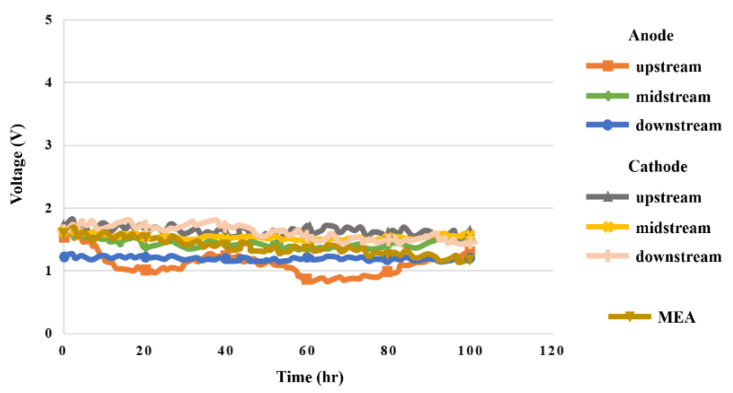
The 100 h voltage variation inside the high-pressure PEMWE.

The high-pressure PEMWE in this study was measured under constant voltage 1.8 V for 100 h. The signals were captured at intervals of 30 min. The voltage data of the upstream, midstream, and downstream of the anode and cathode and the MEA were measured, as shown in Figure 10. The change at the water inlet in the upstream of the anode was relatively drastic. The cathode had higher voltage than the anode due to higher pressure. It was found after 100 h measurement that the MEA voltage dropped slightly.

### 5.4. Current in High-Pressure PEMWE

**Figure 11:**
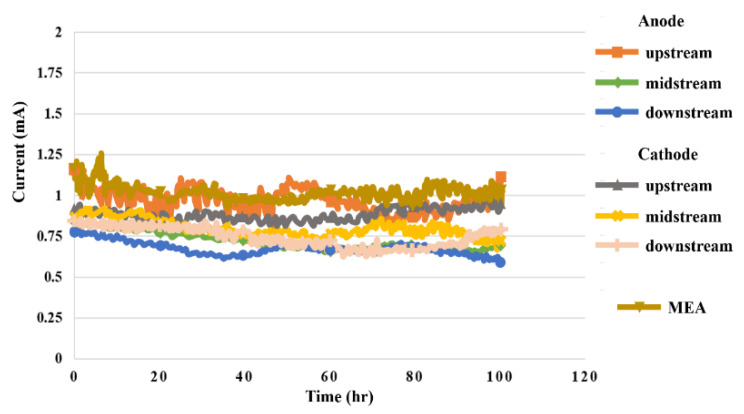
The 100 h current variation inside the high-pressure PEMWE.

The 100 h measurement was performed in an environment of 25 °C, flow rate of 80 mL/min, and constant voltage of 1.8 V. Signals were captured at 30 min intervals. Upstream, midstream, and downstream current data for the anode and cathode and MEA are shown in Figure 11. It can be seen that the current fluctuation at the water inlet upstream of the anode is relatively clear in the reaction process.

### 5.5. Temperature in High-Pressure PEMWE

**Figure 12:**
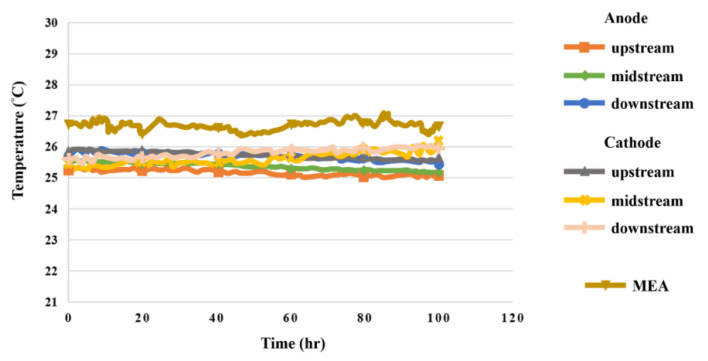
The 100 h temperature variation inside the high-pressure PEMWE.

The temperature monitoring data are shown in Figure 12. In the reaction process of the high-pressure PEMWE, the water in the high-pressure PEMWE generated heat after the reaction, so the temperature change upstream of the anode was relatively large. Although the temperature change between midstream and downstream was small, the temperature was high. With the conduction of water, the temperature at the downstream increased slightly, and the heat was converted into electric energy. The temperature of the MEA was higher, and the variation was larger.

### 5.6. Humidity in High-Pressure PEMWE

**Figure 13:**
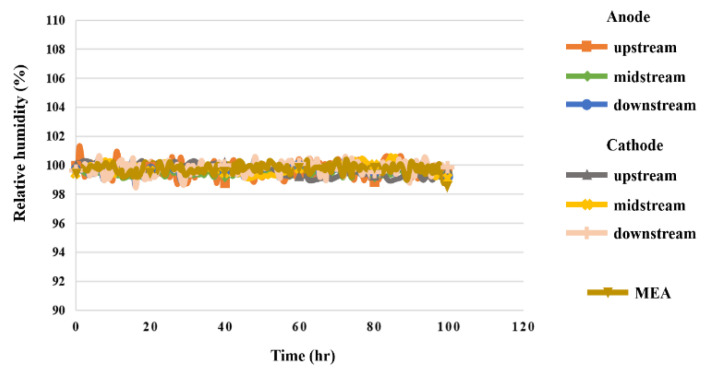
The 100 h humidity variation inside the high-pressure PEMWE.

According to the data in Figure 13, the humidity in the upstream, midstream, and downstream of the anode and cathode was 100% during the reaction of the high-pressure PEMWE, whereas the resistive micro humidity sensor had errors due to slight temperature changes, but the humidity measured through the MEA showed good water permeability; there was no aging or damage.

### 5.7. Pressure in High-Pressure PEMWE

**Figure 14:**
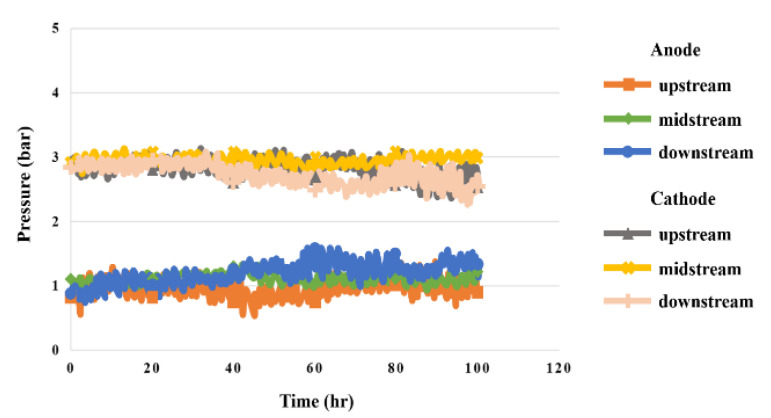
The 100 h pressure variation inside the high-pressure PEMWE.

Figure 14 shows the pressure distribution in the upstream, midstream, and downstream of the anode and cathode during 100 h test for the high-pressure PEMWE. The pressure was measured with 3 bar pressure given at the cathode outlet, and the anode pressure was kept higher than 1 atm. If there was leakage inside the high-pressure PEMWE, the internal pressure would clearly change. The experimental results show that the high-pressure PEMWE was well-designed and assembled, and there was no leakage. The bubbles on the MEA can be reduced by appropriate pressure. the performance of the high-pressure PEMWE was improved.

### 5.8. Flow in High-Pressure PEMWE

**Figure 15:**
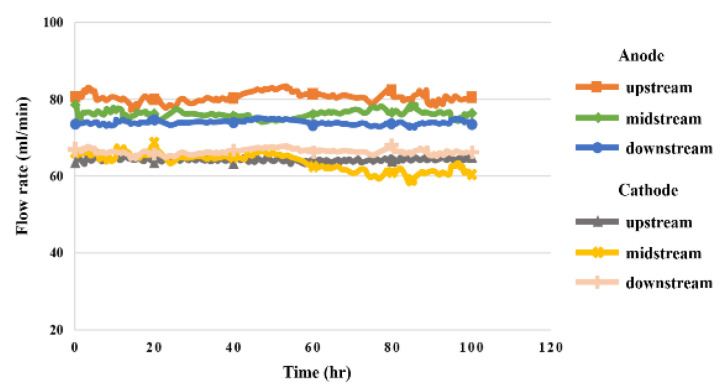
The 100 h flow rate variation inside the high-pressure PEMWE.

The flow distribution in the upstream, midstream, and downstream of the anode and cathode during the 100 h test for the high-pressure PEMWE is shown in Figure 15. It was observed that the flow velocity at the upstream water inlet of anode flow channel was the highest, and that at the downstream water outlet was the lowest because the fluid was smoother and steadier in the columnar channel of the PEMWE than in the serpentine flow channel. The flow velocity at the cathode was lower than that at the anode, because the fluid flowed through the MEA.

### 5.9. Hydrogen in High-Pressure PEMWE

**Figure 16:**
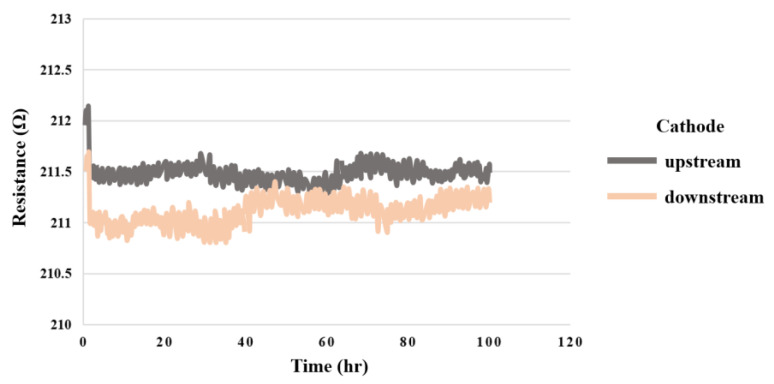
The 100 h hydrogen variation inside the high-pressure PEMWE.

Figure 16 shows the results of 100 h measurement of the high-pressure PEMWE at the two hydrogen flow channel outlets in the upstream and downstream of the cathode. However, since the microhydrogen sensor could not touch oxygen to restore it to the state before the measurement, it could only detect a significant drop in resistance when hydrogen was produced and detect that hydrogen had begun to be produced.

**Figure 17:**
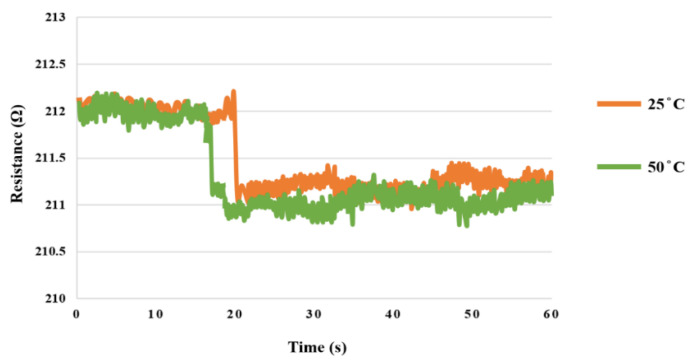
Hydrogen production rates at 25 °C and 50 °C.

In this study, the microhydrogen sensor was placed at the hydrogen production outlet in the downstream of the cathode and measured under 1.8 V constant voltage. The hydrogen production rates were compared using 25 °C and 50 °C DI water, respectively. There was hydrogen generated at about 19 s in the case of 25 °C, and at about 15 s in the case of 50 °C. Therefore, hydrogen production rate at 50 °C was higher than that at 25 °C, as shown in Figure 17.

### 5.10. Relationships among Internal Physical Quantities of High-Pressure PEMWE during 100 h

**Figure 18:**
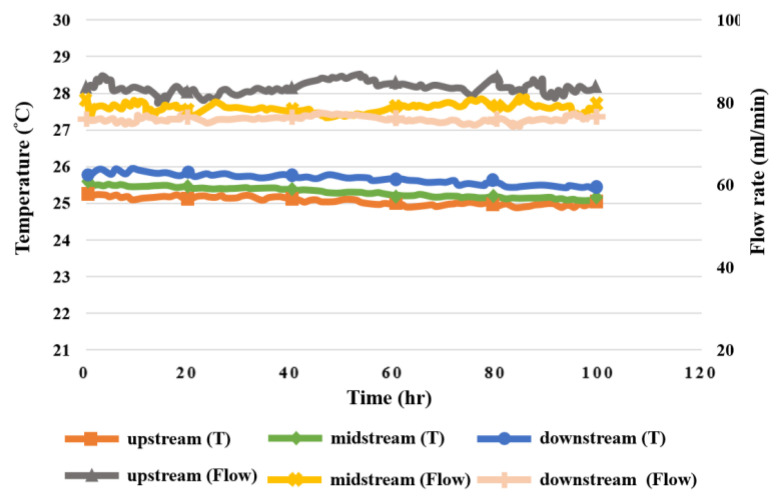
Graph of relation between temperature and flow inside the anode of the high-pressure PEMWE within 100 h.

**Figure 19:**
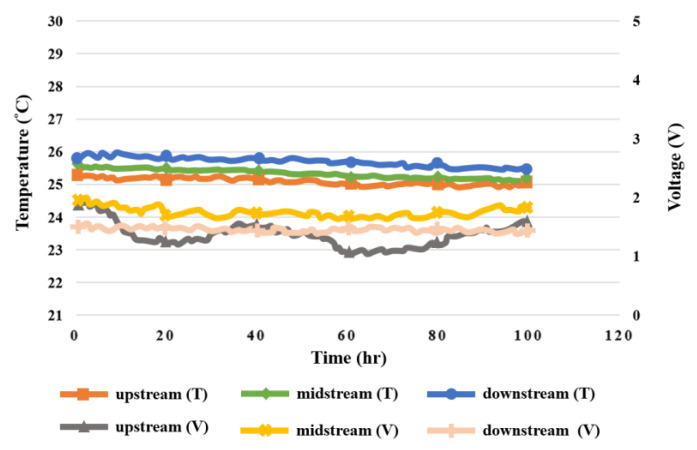
Graph of relation between temperature and voltage inside the anode of the high-pressure PEMWE within 100 h.

**Figure 20:**
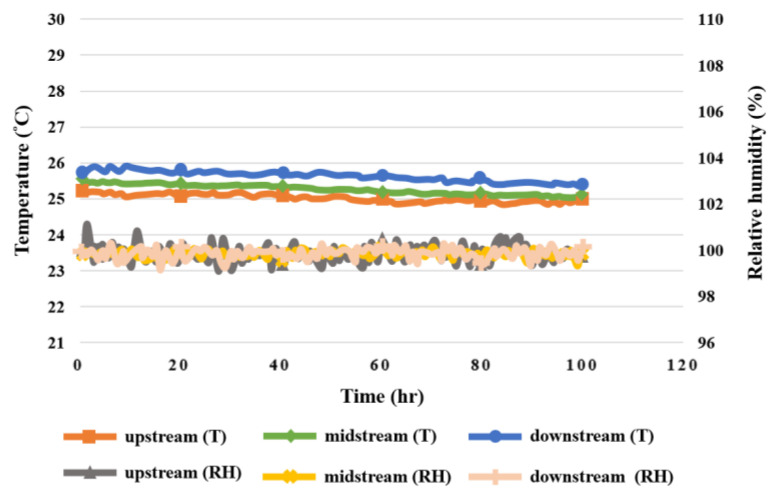
Graph of relation between temperature and humidity inside the anode of the high-pressure PEMWE within 100 h.

**Figure 21:**
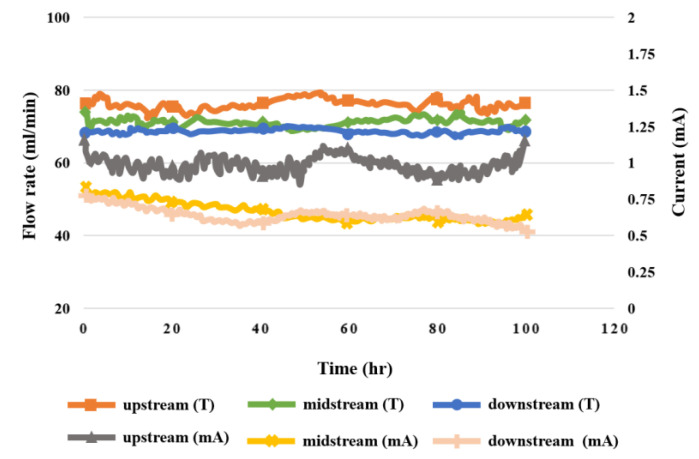
Graph of relation between flow and current inside the anode of the high-pressure PEMWE within 100 h.

Taking the test for the high-pressure PEMWE in the upstream, midstream, and downstream of the anode in the operating conditions of 25 °C operating temperature and 1.8 V constant voltage as an example, the results show that the internal heat distribution was not completely uniform during the operation of the high-pressure PEMWE, and the heat generated by the electrochemical reaction accumulated in the water electrolyzer, so that the temperature change in the upstream and downstream of the anode was relatively smooth, but the temperatures were relatively high. According to the temperature and flow monitoring data, the changes in the upstream, midstream, and downstream were slight, meaning the inside of the high-pressure PEMWE was stable. The temperature- and pressure-monitoring data indicated that the change in upstream water inflow was relatively dramatic. Generally, the higher the temperature, the lower the relative humidity. However, the temperature change during the working process of the high-pressure PEMWE was very small, and the environment was filled with water, so there was no significant correlation between them according to the temperature- and humidity-monitoring data. Figure 18, Figure 19 and Figure 20 show the relationship between temperature, flow, voltage, and humidity upstream, midstream, and downstream of the anode in the high-pressure PEMWE. According to the data plot of flow and current, the current can affect the flow of the fluid. The current change at the upstream inlet of the anode is relatively drastic and the flow rate change is larger, as shown in Figure 21.

## 6. Conclusions

In this study, we successfully embedded seven sets of flexible seven-in-one microsensors upstream, midstream, and downstream of the anode and cathode of the high-pressure PEMWE and MEA without affecting the operation of the high-pressure PEMWE. Internal and local voltage, current, temperature, humidity, pressure, flow, and hydrogen data of the high-pressure PEMWE were successfully collected by the NI PXI 2575 data acquisition unit and LCR meter during the 100 h operating period of the high-pressure PEMWE. The high-pressure PEMWE was not damaged and did not fail during the 100 h operation, and the hydrogen production rate was higher at a high temperature (50 °C).

## Data Availability

Not applicable.
